# Chronic rhinosinusitis possibly associated with decreased lung function in chronic cough patients

**DOI:** 10.1016/j.bjorl.2024.101424

**Published:** 2024-03-21

**Authors:** Pengfei Zhao, Shin Kariya, Takaya Higaki, Seiichiro Makihara, Toru Rikimaru, Mitsuhiro Okano, Mizuo Ando

**Affiliations:** a Okayama University Academic Field of Medicine, Dentistry and Pharmaceutical Sciences, Department of Otolaryngology-Head and Neck Surgery, Okayama, Japan; b Kawasaki Medical School, Department of Otolaryngology-Head and Neck Surgery, Kurashiki, Japan; c Fukuoka Sanno Hospital, Division of Respiratory Medicine, Fukuoka, Japan; d International University of Health and Welfare School of Medicine, Department of Otorhinolaryngology, Narita, Japan

**Keywords:** Asthma, Sinusitis, Rhinitis, Pulmonary function, Cough

## Abstract

**Objectives:**

The purpose of this study is to investigate the lung function in Chronic Rhinosinusitis (CRS) patients with Chronic Cough (CC).

**Methods:**

A total of 1413 CC patients were retrospectively screened and 109 CRS patients with CC were enrolled. Lung function, Lund-Mackay Computed Tomography (CT) score, smoking status, peripheral blood eosinophil count, and immunoglobulin E concentration in serum samples, and Sino-Nasal Outcome Test were examined. Normal control subjects are also recruited.

**Results:**

The Forced Expiratory Volume in 1 second (FEV_1.0_), Percent Predicted FEV_1.0_, and FEV_1.0_/Forced Vital Capacity (FVC) ratio in the patients were significantly low as compared with the control subjects. The FEV_1.0_/FVC ratio was negatively correlated with the Lund-Mackay CT scores of the patients with a high CT score.

**Conclusions:**

The CRS patients with CC should be investigated with lung function. In addition, the multidisciplinary evaluation including a pulmonologist is needed to manage the CRS patients with CC.

**Level of evidence:**

Level 4.

## Highlights

• The chronic rhinosinusitis patients with chronic cough had significant obstructive lung function.
• The SNOT score is high in chronic cough patients with chronic rhinosinusitis.
• The lower airway examination is important in chronic cough patients.

## Introduction

A close relationship between upper and lower airway diseases is well known, and the “United Airways Concept” has been widely accepted.^1^ Chronic cough (CC) is a common illness. Approximately 10–20% of the general population may suffer from this condition, resulting in significant impairment of quality of life.^2^ CC is defined as a persistent cough lasting longer than eight weeks, and numerous factors including asthma, Chronic Obstructive Pulmonary Disease (COPD), gastroesophageal reflux disease, tobacco smoking, occupational irritants, and air pollution have a significant impact both in the onset and development of CC.^3^

Chronic Rhinosinusitis (CRS) is a heterogeneous and complex disease of the nose and paranasal sinuses that occurs in 1–5% of the population of the United States of America.^4^ Cough is one of the important symptoms of CRS, and a possible relationship between CRS and CC has been reported.^5^, ^6^ CRS might also have some impact on the pathogenesis of lung diseases related to CC;^7^ however, a definitive mechanism has not been revealed.

To the best of our knowledge, no previous study has examined the pulmonary function in CRS patients with CC diagnosed by the European Position Paper on Rhinosinusitis and Nasal Polyps (EPOS) criteria.^8^ The purpose of this study is to reveal the lung condition in CRS patients with CC who have no abnormal findings on radiological chest examination.

## Methods

### Subjects

**Figure 1:**
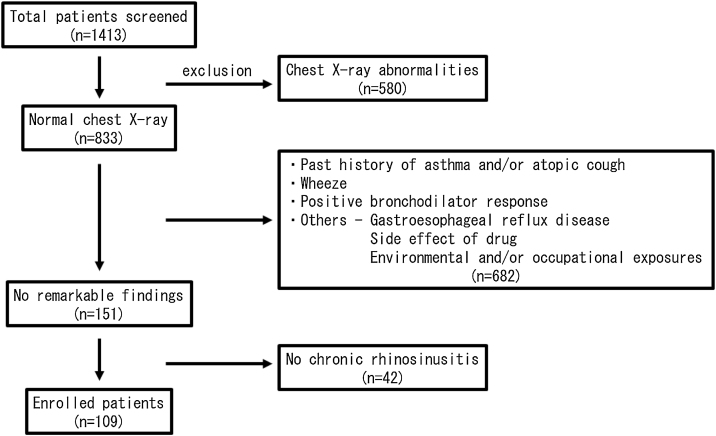
Flow chart of main study participants in the case-control study.

This is a retrospective case-control study. A total of 1413 CC patients visiting in our hospital between 2010 and 2015 were enrolled in this study. All of them received medical examinations by respiratory physicians. The patients were carefully interviewed and were examined by chest X-Ray, bronchial obstruction reversibility test using a short-acting β2 adrenergic receptor agonist, and pulmonary function test. Then, the patients with wheeze, a positive bronchodilator response and/or chest X-Ray abnormalities were excluded from the study. In addition, the patients with gastroesophageal reflux disease were also excluded by frequency scale for the symptoms of gastroesophageal reflux disease questionnaire, a validated questionnaire in the Japanese population.^9^ A paranasal sinus Computed Tomography (CT) scan was performed on all patients, and the patients with CRS based on the EPOS criteria^8^ were selected. In the end, 109 patients were left to be evaluated (Fig. 1). The 53 volunteers without any chronic respiratory symptoms were also recruited in our hospital. Because 3 subjects were excluded from this study because of the past history of asthma, 50 subjects were enrolled as the age/gender-matched normal control participants. Informed consent was obtained from the enrolled subjects. The study was approved by the Institutional Review Board (IRB approval number: FS-79) and conducted in compliance with the Declaration of Helsinki.

### Pulmonary function test

Pulmonary function testing was performed (DISCOM-21 FX III, CHEST Inc., Tokyo, Japan) in the afternoon, and the following parameters were measured or calculated: percent predicted Vital Capacity (%VC), Forced Vital Capacity (FVC), Forced Expiratory Volume in 1 second (FEV_1.0_), Percent Predicted FEV_1.0_ (%FEV_1.0_), and FEV_1.0_/FVC ratio.^10^ A nose clip was used during the examination. The examination acceptability and reproducibility criteria were adopted according to the guideline.^10^ The reference equation provided by manufacturer was used to define the limits of normality. The examination was done by some technicians receiving the same training. The patients with FEV_1.0_/FVC ratio < 70% were considered having an obstructive respiratory ventilation disorder. The inhaled medication restrictions and tobacco abstinence for one day were required for patients.

### Lund-Mackay CT score

A paranasal sinus CT scan was applied to all participants, and the radiographic severity of CRS was assessed by the Lund-Mackay CT staging system.^11^

### Peripheral blood test

To assess the atopic status, the following blood tests were used. The white blood cell and eosinophil counts were measured in peripheral blood. Eosinophil counts > 500/μL was considered as eosinophilia. The total Immunoglobulin E (IgE) in serum samples (normal value < 170 IU/mL) was also determined by contractor (BML Inc., Tokyo, Japan). These tests were performed at the same time.

### Bronchodilator reversibility test

To exclude the subclinical asthmatic patients, a bronchial obstruction reversibility test was applied to all CC patients according to guideline.^10^ Thirty minutes after inhalation of a bronchodilator using nebulizer (salbutamol, 5 mg/mL, 0.3 mL), the pulmonary function test was performed. The increase of FEV_1.0_ ≥ 12% and 200 mL from baseline after inhalation of a short-acting β2-agonist was considered as positive in this test.

### Sino-Nasal Outcome Test (SNOT-20)

The Sino-Nasal Outcome Test (SNOT-20) is a useful questionnaire designed to evaluate subjective symptoms.^12^ SNOT-20 (need to blow the nose, sneezing, runny nose, cough, postnasal discharge, thick nasal discharge, ear fullness, dizziness, ear pain, facial pain/pressure, difficulty falling asleep, waking up at night, lack of a good night’s sleep, waking up tired, fatigue, reduced productivity, reduced concentration, frustrated/restless/irritable, sad, and embarrassed) was used to assess the severity of an individual's symptoms in CC patients.

### Statistical analysis

Data are presented as mean ± standard deviation. A Chi-Square test was used to determine any difference between proportions. A non-parametric statistical method (Mann-Whitney *U* test) was used for comparisons between the two groups. Spearman's rank correlation coefficient was applied to correlation analysis to study the strength of a relationship between the two groups. A *p-*value less than 0.05 was regarded as statistically significant. Statistical analyses were performed using commercially available software (IBM SPSS Statistics, Version 24, IBM, New York, USA).

## Results

The clinical characteristics of the CRS patients with CC and the normal control subjects are summarized in Table 1. There was no significant difference between the groups in age, gender, and smoking status. An abnormal soft tissue shadow without any respiratory symptoms in the paranasal sinus was detected on CT in some control subjects. There was a significant difference in eosinophil count in peripheral blood between the groups.

**Table 1:** Characteristics of the patients and the normal control subjects.

	Patients	Controls	*p*
Number of subjects (n)	109	50	
Male/Female (n)	57 / 52	27 / 23	0.837
Age (years)	50.1 ± 14.8	47.9 ± 13.0	0.357
Height (cm)	164.7 ± 9.3	162.8 ± 9.4	0.292
Body weight (kg)	63.2 ± 13.8	62.7 ± 17.1	0.422
Body mass index	23.2 ± 4.1	23.4 ± 4.8	0.922
Lund-Mackay CT score	9.1 ± 4.5	0.9 ± 1.7	<0.001
Smoking status			
Ex-smoker (n)	38	9	
Current smoker (n)	14	10	0.080
Never smoker (n)	57	31	
Brinkman index			
Ex-smoker	278.8 ± 321.7	325.0 ± 159.5	0.107
Current smoker	463.9 ± 246.3	532.5 ± 309.1	0.578
Peripheral blood examination			
White blood cell count (/µL)	6,433.5 ± 1,714.7	5,984.0 ± 1,572.3	0.130
Eosinophil count (/µL)	309.8 ± 257.1	153.5 ± 126.1	<0.001
Eosinophil count (%)	5.0 ± 4.2	2.6 ± 2.0	<0.001
Serum IgE level (IU/mL)	229.2 ± 387.3	112.7 ± 114.8	0.922

CT, Computed Tomography.

Data represent mean ± standard deviation.

Table 2 shows the pulmonary function in the CRS patients with CC and the normal control subjects. There was no statistically significant difference in %VC between the CRS patients with CC and the normal control subjects. In contrast, the FEV_1.0_/FVC ratio in the CRS patients with CC (median, 78.9%) was significantly lower than that in the normal control subjects (median, 83.9%) (*p* <  0.05). The FEV_1.0_/FVC ratio is the most accepted parameter for showing obstructive lung function changes in the pulmonary function test. In addition, other parameters (FEV_1.0_ and %FEV_1.0_) also indicating obstructive lung function changes in the CRS patients with CC were significantly lower than the respective parameters for the normal control subjects (*p* <  0.05). There was no significant difference in pulmonary function between before and after the bronchodilator inhalation in the CRS patients with CC. There was no significant difference in %VC and FVC between smoker (*n* = 52) and non-smoker (*n* = 57) in the CRS patients with CC (*p* >  0.05). The non-smoker patients showed significantly better lung function (FEV_1.0_/FVC ratio, %FEV_1.0_, and FEV_1.0_) compared to those with smoke (*p* <  0.05).

**Table 2:** Pulmonary function in the patients before bronchodilator inhalation and the normal control subjects.

	Patients	Controls	*p*
%VC (%)	95.0 ± 11.9	94.4 ± 10.0	0.898
FVC (L)	3.35 ± 0.89	3.45 ± 0.86	0.397
FEV_1.0_ / FVC ratio (%)	77.9 ± 7.9	82.5 ± 11.7	<0.001
%FEV_1.0_ (%)	87.7 ± 13.0	96.9 ± 10.6	<0.001
FEV_1.0_ (L/s)	2.61 ± 0.74	2.89 ± 0.69	0.024

Data represent mean ± standard deviation.

%VC, Percent Predicted Vital Capacity; FVC, Forced Vital Capacity; FEV_1.0_, Forced Expiratory Volume in 1 second; %FEV_1.0_, Percent Predicted FEV_1.0_.

**Figure 2:**
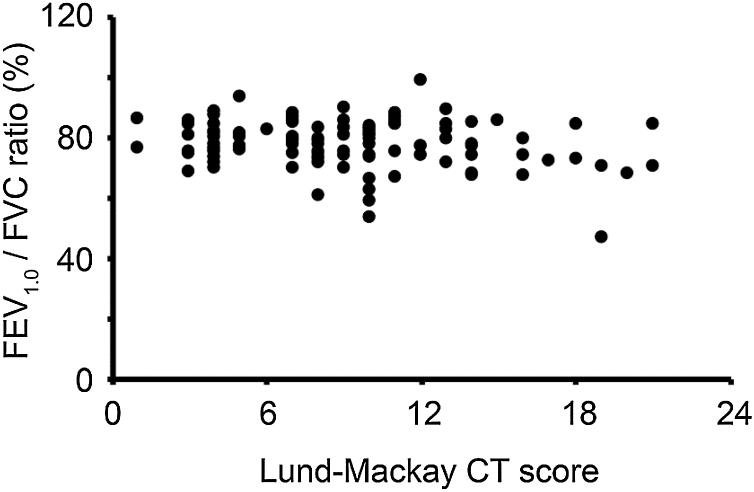
Relationship between the Forced Expiratory Volume in 1 second (FEV1.0)/Forced Vital Capacity (FVC) ratio (FEV1.0/FVC ratio) before bronchodilator inhalation and the Lund-Mackay Computed Tomography (CT) score of chronic cough patients with normal chest X-Ray findings.

In the Lund-Mackay CT staging system, scores can range between 0 and 24. We divided the CRS patients with CC into two groups based on the CT score (low CT score group, Lund-Mackay CT score < 12; high CT score group, Lund-Mackay CT score ≥12). The FEV_1.0_/FVC ratio in the high CT score group (76.2% ± 9.6%) was lower than that in the low CT score group (78.5% ± 7.3%); however, the difference did not reach statistical significance. The correlation analysis showed no notable significant correlation between the FEV_1.0_/FVC ratio and Lund-Mackay CT scores of the CRS patients with CC who had a low CT score (<12). In contrast, the FEV_1.0_/FVC ratio of the patients with a high CT score (≥12) negatively correlated with their Lund-Mackay CT scores (*p* <  0.05) (Fig. 2).

The SNOT-20 scores are shown in Table 3. The scores for postnasal discharge and thick nasal discharge in the patients with a high CT score (≥12) were significantly higher than the respective scores of the patients with a low CT score (<12) (*p* <  0.05). The scores in the rhinologic domain of SNOT-20 (need to blow the nose, sneezing, runny nose, postnasal discharge, and thick nasal discharge)^13^ in the patients with a high CT score (≥12) (10.7 ± 5.5) were significantly higher than those in the patients with a low CT score (<12) (7.8 ± 5.1) (*p* <  0.05). The scores in the rhinologic domain of SNOT-20 in patients with a high CT score (≥12) and FEV_1.0_/FVC ratio tended to correlate but did not reach statistical significance. There was no significant correlation between the SNOT-20 scores (ear and facial symptoms domain, sleep domain, and psychological domain) and lung function.

**Table 3:** SNOT scores of the patients with chronic cough.

	CT score < 12	CT score ≥ 12	*p*
Total	25.3 ± 14.7	27.2 ± 16.8	0.569
Need to blow nose	2.3 ± 1.5	2.9 ± 1.5	0.120
Sneezing	1.3 ± 1.2	1.0 ± 1.2	0.307
Runny nose	1.7 ± 1.7	2.2 ± 1.8	0.258
Cough	3.5 ± 1.4	3.4 ± 1.0	0.509
Postnasal discharge	1.4 ± 1.5	2.5 ± 1.6	0.012
Thick nasal discharge	1.2 ± 1.6	2.2 ± 1.7	0.017
Ear fullness	1.0 ± 1.5	1.3 ± 1.5	0.152
Dizziness	0.7 ± 1.2	0.5 ± 1.1	0.209
Ear pain	0.3 ± 0.8	0.4 ± 1.0	0.835
Facial pain/pressure	0.6 ± 1.3	0.8 ± 1.5	0.839
Difficulty falling asleep	1.6 ± 1.8	1.6 ± 1.8	0.776
Waking up at night	2.3 ± 1.9	2.1 ± 1.8	0.595
Lack of good night’s sleep	2.4 ± 1.8	2.0 ± 1.7	0.487
Waking up tired	2.1 ± 1.6	1.9 ± 1.4	0.876
Fatigue	2.6 ± 1.6	2.3 ± 1.5	0.537
Reduced productivity	1.7 ± 1.5	1.6 ± 1.5	0.810
Reduced concentration	1.8 ± 1.5	1.6 ± 1.3	0.758
Frustrated/restless/irritable	1.6 ± 1.4	1.5 ± 1.5	0.748
Sad	1.1 ± 1.4	0.8 ± 1.1	0.575
Embarrassed	2.7 ± 1.5	2.9 ± 1.4	0.695

Data represent mean ± standard deviation.

SNOT, Sino-Nasal Outcome Test; CT, Computed Tomography.

To exclude the effect of smoke, next we show the findings in never smokers only. The characteristics of the CRS patients with CC and the normal control subjects who never smoke are summarized in Table 4. The CRS patients with CC had high eosinophil counts as compared with control subjects, however the mean eosinophil counts in patients’ group was < 500/μL. The mean levels of total IgE in serum samples were within normal value (< 170 IU/mL) in both groups. There was no significant difference in the level of IgE between CRS patients with CC and control subjects.

**Table 4:** Characteristics of the patients and the normal control subjects who never smoke.

	Patients	Controls	*p*
Number of subjects (n)	57	31	
Male/Female (n)	15 / 42	13 / 18	
Age (years)	48.8 ± 15.6	48.8 ± 14.5	0.903
Height (cm)	161.4 ± 9.6	160.2 ± 8.6	0.937
Body weight (kg)	60.0 ± 15.4	60.4 ± 14.3	0.830
Body mass index	22.9 ± 4.4	23.4 ± 4.5	0.567
Lund-Mackay CT score	8.5 ± 4.6	1.1 ± 1.9	<0.001
Peripheral blood examination			
White blood cell count (/µL)	6,505.5 ± 1,867.9	5,849.0 ± 1,739.5	0.122
Eosinophil count (/µL)	313.3 ± 278.4	138.2 ± 133.1	0.002
Eosinophil count (%)	4.9 ± 4.5	2.3 ± 2.1	0.003
Serum IgE level (IU/mL)	141.2 ± 176.1	90.3 ± 71.0	0.840

CT, Computed Tomography.

Data represent mean ± standard deviation.

Table 5 shows the pulmonary function in the CRS patients with CC and the normal control subjects who never smoke. There was no statistically significant difference in %VC between the CRS patients with CC and the normal control subjects. In contrast, the FEV_1.0_/FVC ratio in the CRS patients with CC (median, 80.5%) was significantly lower than that in the normal control subjects (median, 84.2%) (*p* <  0.05). In addition, FEV_1.0_ and %FEV_1.0_ in the CRS patients with CC were significantly lower than the normal control subjects, respectively (*p* <  0.05).

**Table 5:** Pulmonary function in the patients before bronchodilator inhalation and the normal control subjects who never smoke.

	Patients	Controls	*p*
%VC (%)	95.5 ± 12.0	94.5 ± 10.8	0.841
FVC (L)	3.08 ± 0.94	3.28 ± 0.86	0.196
FEV_1.0_/FVC ratio (%)	79.5 ± 6.1	81.5 ± 14.4	0.006
%FEV_1.0_ (%)	90.4 ± 12.4	98.6 ± 12.6	0.010
FEV_1.0_ (L/s)	2.45 ± 0.77	2.75 ± 0.72	0.045

Data represent mean ± standard deviation.

%VC, Percent Predicted Vital Capacity; FVC, Forced Vital Capacity; FEV_1.0_, Forced Expiratory Volume in 1 second; %FEV_1.0_, Percent Predicted FEV_1.0_.

There was no significant difference in FEV_1.0_/FVC ratio between the high CT score group and the low CT score group in CRS patients with CC who never smoke. No significant correlation was observed between the FEV_1.0_/FVC ratio and Lund-Mackay CT scores of the CRS patients with CC who never smoke.

The SNOT-20 scores in CRS patients with CC who never smoke are shown in Table 6. The scores for postnasal discharge in the patients with a high CT score (≥12) were significantly higher than that in the patients with a low CT score (<12) (*p* <  0.05).

**Table 6:** SNOT scores of the patients with chronic cough who never smoke.

	CT score < 12	CT score ≥ 12	*p*
Total	26.3 ± 12.7	28.7 ± 14.2	0.682
Need to blow nose	2.2 ± 1.6	2.8 ± 1.5	0.356
Sneezing	1.4 ± 1.2	0.8 ± 1.0	0.316
Runny nose	1.9 ± 1.6	3.0 ± 2.0	0.185
Cough	3.7 ± 1.2	3.7 ± 1.2	0.830
Postnasal discharge	1.2 ± 1.4	3.5 ± 1.0	0.002
Thick nasal discharge	1.2 ± 1.6	2.2 ± 1.9	0.185
Ear fullness	1.1 ± 1.6	1.3 ± 1.5	0.711
Dizziness	0.7 ± 0.9	0.5 ± 1.2	0.571
Ear pain	0.4 ± 0.8	0.2 ± 0.4	0.625
Facial pain/pressure	0.8 ± 1.3	1.2 ± 1.5	0.625
Difficulty falling asleep	1.9 ± 1.7	1.5 ± 1.9	0.598
Waking up at night	2.2 ± 1.7	2.3 ± 2.0	0.984
Lack of good night’s sleep	2.2 ± 1.7	1.7 ± 1.8	0.445
Waking up tired	1.8 ± 1.5	2.0 ± 1.4	0.682
Fatigue	2.8 ± 1.4	2.3 ± 1.4	0.598
Reduced productivity	1.6 ± 1.5	1.3 ± 1.5	0.711
Reduced concentration	1.8 ± 1.5	1.5 ± 1.4	0.711
Frustrated/restless/irritable	1.6 ± 1.5	1.7 ± 1.6	0.984
Sad	1.1 ± 1.2	0.8 ± 1.6	0.399
Embarrassed	2.7 ± 1.5	3.7 ± 1.0	0.159

Data represent mean ± standard deviation.

SNOT, Sino-Nasal Outcome Test; CT, Computed Tomography.

## Discussion

The prevalence of CRS is very high throughout the world, and patients with CRS have impaired quality of life.^14^ CRS might affect the lower airway.^15^ Some studies reported that the patients with CRS had obstructive lung function changes, and intensive management for CRS could improve lower airway diseases.^16^, ^17^, ^18^, ^19^, ^20^ On the other hand, no significant effect of treatment of CRS on lung diseases has been reported.^21^ How to manage CRS in patients with lower airway diseases has been controversial, and respiratory physicians tend to think lightly of the presence of upper airway diseases.^22^

The pathogenesis of CC is complex. A recent study reported that most CC patients had abnormal findings on radiological chest examination, but CRS might be considered as one cause of CC.^5^, ^7^ In addition, low-dose, long-term macrolide antibiotics treatment could improve lung function in CRS patients with CC who had normal chest X-Ray findings.^6^ In this study, the physical examination, chest X-Ray, and pulmonary function test on all patients were done by respiratory physicians, and the patients with subjective symptom of asthmatic cough, wheeze and any abnormal findings of bronchodilator reversibility test were excluded from the study. Our findings showed that CRS patients with CC had obstructive pulmonary function changes as compared with the normal control subjects.

The Lund-Mackay CT scoring system is a useful tool to evaluate radiological disease severity in CRS. Asymptomatic mucosal abnormalities in the paranasal sinus are sometimes found incidentally on CT scan, and several studies have reported findings within the normal range of the Lund-Mackay CT score.^23^, ^24^ In this study, some control subjects without respiratory symptoms had a paranasal sinus abnormality on CT. Because a Lund-Mackay CT score ≤3 may be considered within normal limits, abnormal CT findings in our control group might not have a clinically significant impact. Lung function was affected in the CC patients with a high Lund-Mackay CT score (≥12) in this study, and their CT scores were negatively correlated with the FEV_1.0_/FVC ratio. Especially in patients with severe CRS, CRS may have an undesirable effect on lung function.

Postnasal drip is a possible cause of CC in patients with CRS.^25^, ^26^ We showed in this study that the patients with a high CT score had increased SNOT scores for postnasal discharge and thick nasal discharge in the rhinologic domain. Postnasal discharge are common symptom in smokers. Our findings showed that the never smoker patients with a high CT score also had increased SNOT scores for postnasal discharge. A previous study showed that the concentration of Interleukin-5 (IL-5) in nasal secretions was significantly correlated with pulmonary function test parameters showing obstructive lung function changes in patients with CRS.^19^ IL-5 is a cytokine released from type-2 T helper lymphocytes, type-2 innate lymphoid cells, mast cells, and eosinophils, and is a key cytokine in eosinophil development and activation. In this study, the CRS patients with CC had a higher eosinophil count in peripheral blood as compared with the control subjects, though no significant difference was noted in serum IgE levels between the groups. Our findings suggest that systemic eosinophilia and/or postnasal discharge containing unfavorable factors might have some role in decreased lung function in CC patients.

There are several limitations in this study, including a small sample size and lack of analysis of nasal secretions. In addition, the patients group including smoker had high eosinophil count in peripheral blood and serum IgE level. These findings may show that the patients have potential allergic background, and the results are modified. Further studies are needed to reveal the definitive mechanism of obstructive lung function changes in CRS patients with CC.

## Conclusions

The CRS patients with CC who had no clinically obvious lung disease showed obstructive lung function changes. The FEV_1.0_/FVC ratio decreased especially in the patients with severe CRS. Postnasal drip and nasal discharge might have some role in the affected lung function in the CRS patients with CC. The lower airway should be carefully assessed in CC patients. Treatment for CRS should be considered in patients presenting CC.

## Conflicts of interest

The authors declare no conflicts of interest.
